# Experimental Investigation and Response Surface Optimization of Fiber-Reinforced Polymer Concrete for Mining Roadway Support

**DOI:** 10.3390/ma19184028

**Published:** 2026-09-21

**Authors:** Linlin Wang, Guozhong Liu, Qingming Long, Dawang Zhang, Yiren Wang

**Affiliations:** 1 State Key Laboratory of Coal Mine Disaster Prevention and Control, Chongqing 400039, China; 2 China Coal Technology and Engineering Group Chongqing Research Institute, Chongqing 400039, China; 3 College of Materials Science and Engineering, Xi’an University of Architecture and Technology, Xi’an 710055, China; 4 School of Environment and Civil Engineering, Dongguan University of Technology, Dongguan 523000, China

**Keywords:** fiber-reinforced polymer concrete, response surface optimization, flexural-to-compressive strength ratio, styrene–acrylic emulsion, microstructure

## Abstract

The escalating intensity and depth of coal mining operations have exacerbated underground strata pressure, resulting in fracture-induced air leakage channels that heighten risks of coal spontaneous combustion and gas explosions. These challenges necessitate enhanced flexibility and strength in cementitious materials used for roadway support. This study investigated the mechanical properties of fiber-reinforced polymer concrete (FRPC) for mining applications through response surface methodology (RSM) using Design Expert software. Three critical factors: styrene–acrylic emulsion content (5–15 wt.%), polypropylene fiber length (9–15 mm), and fiber content (0.7–1.1 kg/m^3^), were systematically investigated to establish factor-performance correlations via 3D response surfaces. This study experimentally investigated the effects of styrene–acrylic emulsion content, polypropylene fiber length, and fiber content on the mechanical properties of fiber-reinforced polymer concrete for mining roadway support. Response surface methodology was used as an empirical statistical tool to describe the response trends and factor interactions within the selected experimental range. The regression models developed in this study should therefore be interpreted as local empirical models rather than mechanics-based predictive equations. Using the flexural-to-compressive strength ratio as an index, the optimal formulation of FRPC was 10% emulsion, 12 mm fibers, and 1.1 kg/m^3^ fiber content. Microstructural characterization indicated that polypropylene fibers effectively inhibited crack propagation through bridging effects, while styrene–acrylic emulsion formed continuous film-like network structures on cement surfaces. Synergistically, both components enhanced matrix densification, achieving concurrent improvements in flexibility and strength. Field applications demonstrated that FRPC significantly reduced air leakage channels, decreased the risk of coal spontaneous combustion, and improved the safety of coal mine production.

## 1. Introduction

Coal remains a crucial energy resource and an important component of China’s primary energy system as energy demand continues to increase [1] (Leonard et al., 2018). With the continuous increase in mining depth and mining intensity, the roadway surrounding rock is subjected to more complex concentrated stress and deviatoric stress. These stress conditions can induce deformation, cracking, and damage in surrounding rock and ventilation isolation facilities, thereby forming air leakage channels [2]. Meanwhile, the extension of mining levels generally increases gas content and negative ventilation pressure. Under these conditions, traditional shotcrete concrete used for roadway support often shows high brittleness, limited deformation capacity, and poor gas–liquid sealing performance. Cracking and detachment of the shotcrete layer may further create leakage pathways, increasing the risks of coal spontaneous combustion and gas explosions [3]. Therefore, mining roadway support materials should not only provide sufficient strength but also possess improved flexibility, crack resistance, adhesion, and sealing capacity.

To meet these requirements, polymer modification and fiber reinforcement have been widely considered effective approaches for improving the performance of cement-based materials. Previous studies have shown that incorporating organic polymers or fibers into concrete can improve its mechanical performance and durability [4,5,6]. Compared with conventional concrete, fiber-reinforced concrete can reduce early plastic shrinkage, improve crack resistance, and enhance deformation capacity [7,8]. Organic polymers can also improve the flexibility, adhesion, and impermeability of cement-based materials by modifying the matrix and interfacial transition zones [9]. Carpinteri [10] reported that fiber incorporation can mitigate cracking and improve mechanical performance, while Zheng [11] showed that fiber-reinforced concrete can reduce cracking and shedding during roadway support. In addition, polymer emulsions and rubber powder have been used to improve the mechanical behavior of cement-based materials [12,13]. Water-dispersed polymer emulsions can influence cement hydration and microstructure through adsorption, film formation, and interactions with hydration products, which is beneficial for sprayed concrete used in mining environments [14,15]. Among these polymer modifiers, styrene–acrylic emulsion has the advantages of low cost, wide availability, and convenient application, making it suitable for large-scale mining support. Thus, combining styrene–acrylic emulsion with polypropylene fibers provides a feasible strategy for preparing fiber-reinforced polymer concrete (FRPC) with improved crack resistance, flexibility, and sealing performance [16].

However, the improvement provided by fibers is not determined simply by whether fibers are added. The reinforcing efficiency of fibers depends strongly on fiber content or volume fraction, fiber length, aspect ratio, spatial dispersion, and the interfacial bond between fibers and the cementitious matrix. These parameters directly affect the crack-bridging capacity of fibers, the redistribution of tensile stress across crack surfaces, and the energy required for crack initiation and propagation. An appropriate fiber length and fiber content can delay microcrack development and improve post-cracking deformation capacity, whereas excessive fiber content may cause fiber agglomeration, poor workability, and additional internal defects. From a mechanical perspective, the contribution of fibers is mainly reflected in the post-cracking stage, where fibers bridge cracks and resist crack opening through pull-out, slippage, and interfacial bonding. Established crack-bridging concepts, including the Updated Bridged Crack Model (UBCM), provide a useful framework for understanding the relationship between fiber geometry, fiber dosage, crack opening, and post-cracking response. In this study, UBCM is acknowledged only as a mechanical interpretation framework to explain the roles of fiber length and fiber content; no additional UBCM-based modeling calculation was performed.

In addition to fiber parameters, polymer emulsion content is another key factor affecting the performance of FRPC. Previous studies have shown that styrene–acrylic emulsion can modify cementitious composites through polymer film formation, pore refinement, and improved interfacial bonding among hydration products, aggregates, and fibers [17,18]. An appropriate emulsion content can enhance matrix compactness, crack resistance, and adhesion between the polymer phase and cement hydration products. However, excessive emulsion may reduce matrix stiffness, disturb the continuity of hydration products, or interfere with cement hydration, thereby negatively affecting strength development. Therefore, the mechanical behavior of FRPC is controlled by the coupled effects of polymer emulsion content, fiber length, and fiber content rather than by any single factor. Although previous studies have confirmed the benefits of polymer modification or fiber reinforcement separately, the combined influence of these three variables on the strength, flexibility, microstructure, and field applicability of FRPC for mining roadway support remains insufficiently clarified.

Traditional single-factor experimental methods are limited in revealing such coupled effects because they usually vary one parameter while keeping others unchanged. This approach cannot efficiently identify interactions among polymer emulsion content, fiber length, and fiber content. A full-factorial experimental design would require a large number of tests and would increase experimental complexity [19]. Response surface methodology (RSM) has been widely used as a statistical design and analysis method for evaluating factor interactions and assisting mixture optimization [20,21]. Its applicability in construction materials and related engineering fields has also been demonstrated [22,23]. However, RSM is a mature statistical tool and is not regarded as the novelty of this work.

Therefore, this study experimentally investigates the effects of three selected variables, namely styrene–acrylic emulsion content, polypropylene fiber length, and polypropylene fiber content, on the compressive strength, flexural strength, flexural-to-compressive strength ratio, microstructure, and field applicability of FRPC. RSM was used only to organize the experimental design, evaluate factor interactions, and assist in identifying a suitable mixture within the selected experimental range. The main contribution of this study is to clarify the coupled effects of polymer emulsion and polypropylene fibers on the mechanical behavior, flexibility, and sealing performance of FRPC for mining roadway support, rather than to propose RSM as a new optimization method.

## 2. Experiment

### 2.1. Experimental Materials

Portland cement (P·O 42.5 grade) conforming to the Chinese Standard GB 175-2023 [24] for common Portland cement was used in this experimental work. Coarse aggregate with 5–10 mm particle size and 0.8% powder content was employed. Fine aggregate, obtained from sand with a fineness modulus of 2.4, is characterized in Table 1. The styrene–acrylic emulsion, produced by Dow Chemical Company, featured (50 ± 1)% solid content, pH 7–8, and glass transition temperature (Tg) of 27–28 °C. A Chongqing-manufactured polycarboxylate superplasticizer served as the water reducer, while tributyl phosphate (TBP) functioned as the defoaming agent. The polypropylene (PP) fibers used in this study had a diameter of 32.7 μm and a density of 0.91 g/cm^3^. Their elastic modulus, elongation at break, and tensile strength were 4.236 GPa, 28.4%, and 469 MPa, respectively.

### 2.2. Experimental Design

This study adopted a central composite design (CCD) rather than a full factorial design. Although three factors were investigated at five coded levels, these levels corresponded to the low level (−1), center level (0), high level (+1), and axial levels (−α and +α) in the CCD scheme. Therefore, the five levels listed in Table 2 were not combined in a full factorial manner. A full factorial design would require 5^3^ = 125 runs, whereas CCD can estimate the linear, interaction, and quadratic effects of the selected variables using a reduced number of experiments. In this study, nine experimental runs were designed, including factorial points, axial points, and replicated center points. The replicated center points were used to estimate experimental error and assess the adequacy of the regression model.

### 2.3. Preparation and Testing of Samples

The FRPC mixture proportion consists of cement:water:coarse aggregate:fine aggregate at a ratio of 1:0.55:2.55:2.05. Aggregates and fibers were thoroughly mixed in a mixer before sequentially adding cement, water, and liquid admixtures. After complete mixing, the slurry was poured into 100 × 100 × 100 mm or 100 × 100 × 450 mm molds and compacted using a vibrating table. Specimens were demolded after 24 h and cured in a standard curing room with relative humidity exceeding 95% at 20 ± 2 °C for 28 days. Mechanical properties were evaluated using a microcomputer-controlled electronic pressure testing machine (YAW-300D, Jinan Hensgrand Instrument Co., Ltd., Jinan, China) and a microcomputer-controlled electronic universal testing machine (WDW-50D, Jinan Chuangli Testing Machine CO., Ltd., Jinan, China) The microstructure of specimen cross-sections was observed using scanning electron microscopy (JSM-6700F, Japan Electronics Corporation, Tokyo, Japan) after gold spray treatment. The specimens with dimensions of samples were placed on two cylindrical supports with a clear span of 300 mm, and the load was applied vertically at the mid-span until failure. The loading rate was controlled at approximately 0.05 MPa/s according to the commonly used flexural strength testing procedure for cement-based materials.

## 3. Results and Discussion

Following preliminary exploratory experiments, nine experimental runs were designed using response surface methodology (RSM), and the corresponding experimental results are presented in Table 3. The regression equations established from the CCD results are empirical models intended to describe the response trends and factor interactions within the selected experimental domain. Therefore, these equations were used only for local analysis and mixture optimization in this study.

### 3.1. Effect of Three Factors on the Compressive Strength of Samples

Using Design Expert software, we investigated the significance of styrene–acrylic emulsion and fiber variables, along with their interactions, on compressive and flexural strength. This analysis provides guidance for preparing fiber-reinforced flexible polymer concrete suitable for various conditions and ensures safety in coal mine production. The compressive strength results underwent regression fitting analysis using Design Expert 8.0, yielding a quadratic multiple regression fitting model (Equation (1)).

*R*_1_ = 20.33 − 2.49A + 0.67B + 0.17C − 0.11AB − 0.037AC + 0.021BC + 0.15A^2^ − 0.53B2 − 0.063C^2^ (1)

**Figure 1:**
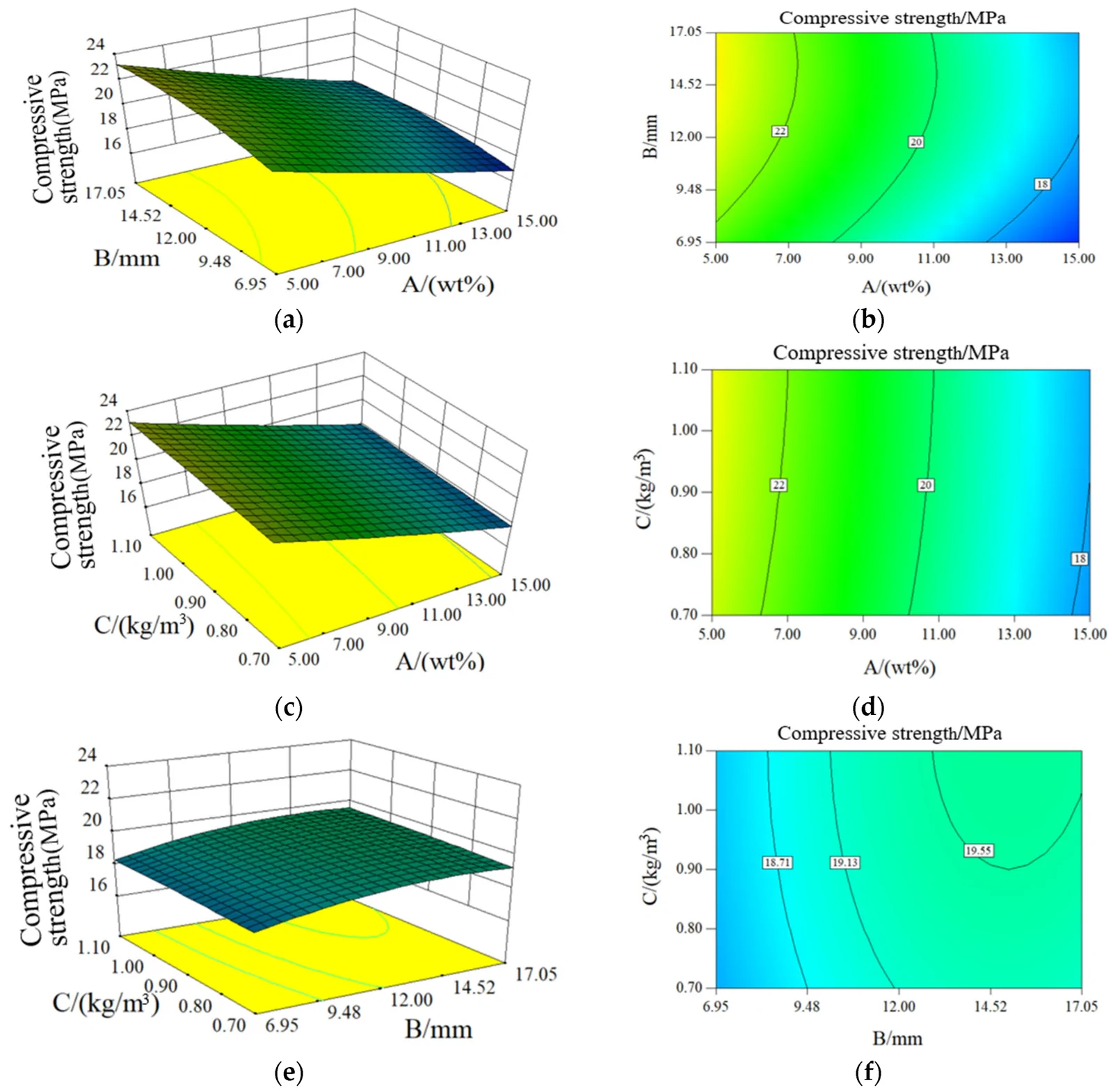
3D response surface (**a**) and contour plots (**b**) of compressive strength: effects of emulsion content A and fiber length B at fixed fiber content C = 0.9 kg/m^3^; 3D response surface (**c**) and contour plots (**d**) of compressive strength: effects of A and C at fixed B = 12 mm; 3D response surface (**e**) and contour plots (**f**) of compressive strength: effects of B and C at fixed A = 10 wt.%.

Statistical analysis in Table 4 confirms the model’s significance with a *p*-value less than 0.0001. The model’s correction coefficient (Adj R-Squared) is 0.9678, and the determination coefficient (R-Squared) is 0.9841. The prediction coefficient (Pred R-Squared) is 0.9087, and the signal-to-noise ratio substantially exceeds 4. These statistical indicators suggest that the fitted model can reasonably describe the compressive strength results within the present experimental domain. However, considering the number of regression terms in the second-order polynomial equation, the model was used only as a local statistical tool for analyzing factor effects and interactions, rather than as a generally applicable predictive model. Figure 1 demonstrates relatively flat response surfaces. The BC and AC response surfaces exhibit similarities, indicating that the interactions between AB and BC on compressive strength are not significant. According to the comprehensive response surface diagrams and contour lines, the significance order of the three factors on compressive strength is emulsion A > fiber content C > fiber dimension length B.

The variation in compressive strength is mainly associated with polymer film formation and fiber incorporation. A moderate amount of styrene–acrylic emulsion can improve interfacial bonding and matrix compactness, whereas excessive emulsion may reduce matrix stiffness and disturb hydration product continuity, leading to lower compressive strength. Compared with flexural behavior, polypropylene fibers have a limited effect on compressive strength because their main role is crack bridging after crack initiation.

### 3.2. Effect of Three Factors on the Flexural Strength of Samples

Based on the flexural strength results in Table 3, a quadratic multiple regression fitting model was developed using Design Expert 8.0, as expressed in Equation (2):

R_2_ = 4.18 + 0.54A + 0.24B + 0.01C − 0.19AB − 0.11AC − 0.11BC − 0.11A^2^ − 0.67B^2^ − 0.006C^2^ (2)

Table 5 confirms the model’s significance with a *p*-value less than 0.0001 and a lack of fit term of 0.2134 > 0.05, indicating a strong correlation between the model and experimental data. The model exhibits a high correction coefficient (Adj R-Squared) of 0.9701, explaining 97.01% of the response value variation. The determination coefficient (R-Squared) is 0.9843, and the prediction coefficient (Pre R-Squared) is 0.9027, both approaching 1. The signal-to-noise ratio is substantial at 30.228, with a C.V.% of 2.39%, demonstrating high experimental reliability and excellent simulation quality. In conclusion, the regression model is reliable, and the regression equation effectively analyzes the experimental results.

**Figure 2:**
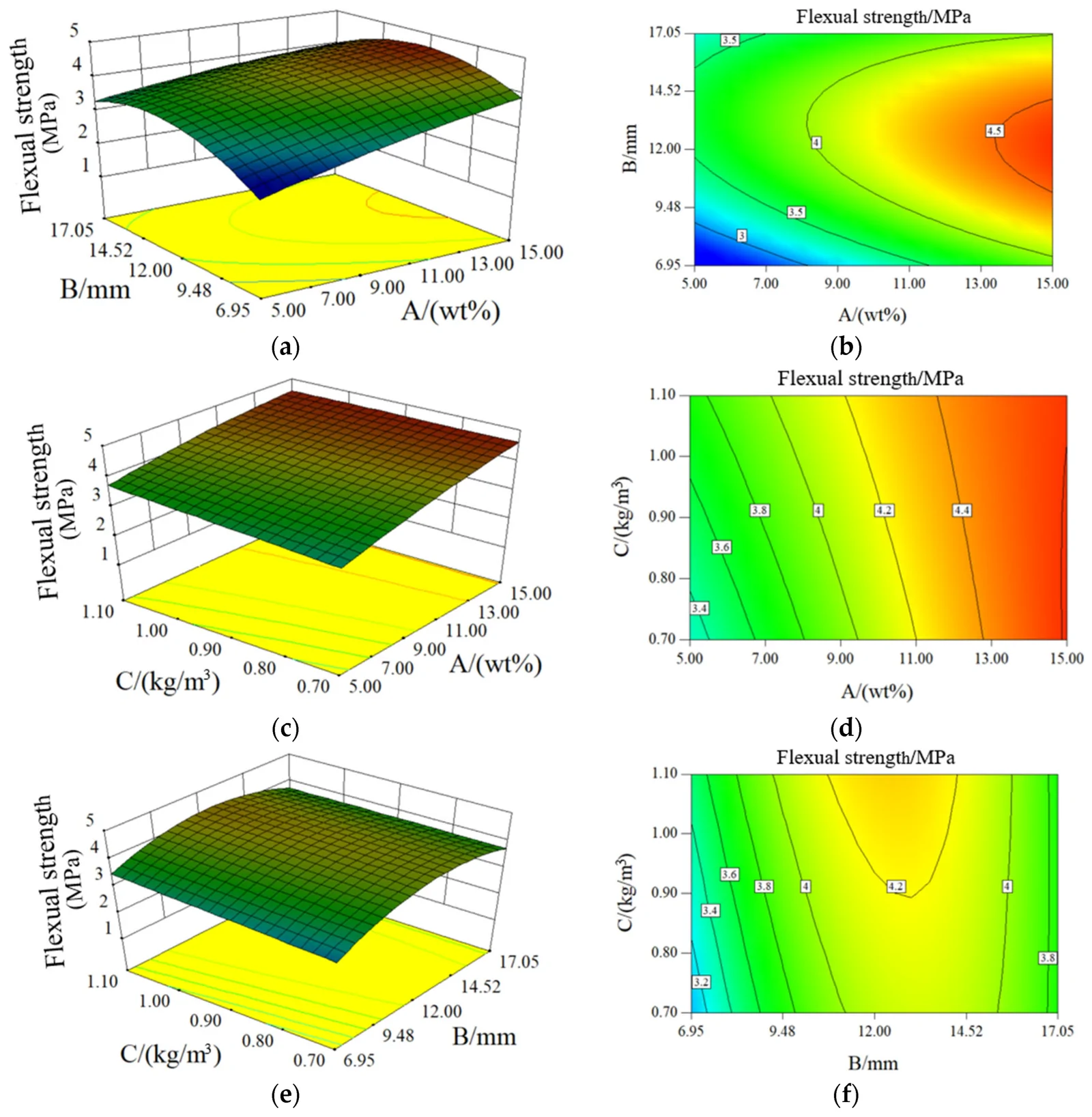
3D response surface (**a**) and contour plots (**b**) of flexural strength: effects of emulsion content A and fiber length B at fixed fiber content C = 0.9 kg/m^3^; 3D response surface (**c**) and contour plots (**d**) of flexural strength:effects of A and C at fixed B = 12 mm; 3D response surface (**e**) and contour plots (**f**) of flexural strength:effects of B and C at fixed emulsion content A = 10 wt.%.

Figure 2 shows that BC and AB response surfaces form downward parabolas, with flexural strength reaching maximum values within the experimental range. Denser contour lines indicate more significant impacts. Variations in emulsion addition have a greater effect on FRPC’s flexural performance. In contrast, the influence of fiber content and fiber length variations is significantly lower, with flexural strength ranging from 2.5 to 2.7 MPa. The relatively steep slope of the response surface curves indicates significant interactions between various factors. The contour line shapes reflect the strength of interaction effects-elliptical contour lines indicate significant interactions between two factors, while mesh or circular contour lines indicate the opposite. The more pronounced distribution of contour lines on the abscissa compared to the ordinate suggests that emulsion A’s influence exceeds that of fiber length C and fiber content B. The significance of interaction magnitudes can be determined by the absolute values of each coefficient in the regression model. In the flexural strength model, the order of significant influence is emulsion A > fiber length B > fiber content C, while the order of significant interaction among factors is AB > AC > BC. The optimal mixture was not determined solely from visual observation of the response surface plots. Numerical optimization was performed using the desirability function in Design Expert, with the flexural-to-compressive strength ratio set to be maximized and the compressive strength constrained to satisfy the basic requirement for mining support concrete.

### 3.3. Effect of Three Factors on the Flexural-to-Compressive Strength Ratio of Samples

Regression fitting analysis was performed on the flexural-to-compressive strength ratio results from Table 3 using Design Expert 8.0, yielding a quadratic multiple regression fitting model as shown in Equation (3):

*R*_3_ = 0.21 + 0.050A − 0.006997B + 0.004160C + 0.008409AB − 0.005AC − 0.004204BC − 0.001307A^2^ − 0.029B^2^ − 0.000461C^2^ (3)

As indicated in Table 6, the highly significant *p* value (*p* < 0.0001) demonstrates the model’s robustness, despite the non-significant lack of fit term (0.2718 > 0.05). The model exhibits excellent statistical parameters, including an adjusted coefficient (Adj R-Squared) of 0.9813, a determination coefficient (R-Squared) of 0.9901, a prediction coefficient (Pred R-Squared) of 0.9450, and a signal-to-noise ratio of 39.447, substantially exceeding the threshold value of 4. With a coefficient of variation of 3.06%, these statistical indicators collectively confirm that the regression model is reliable and appropriate for analyzing the experimental results, providing high confidence in the regression equation’s ability to interpret the experimental data.

**Figure 3:**
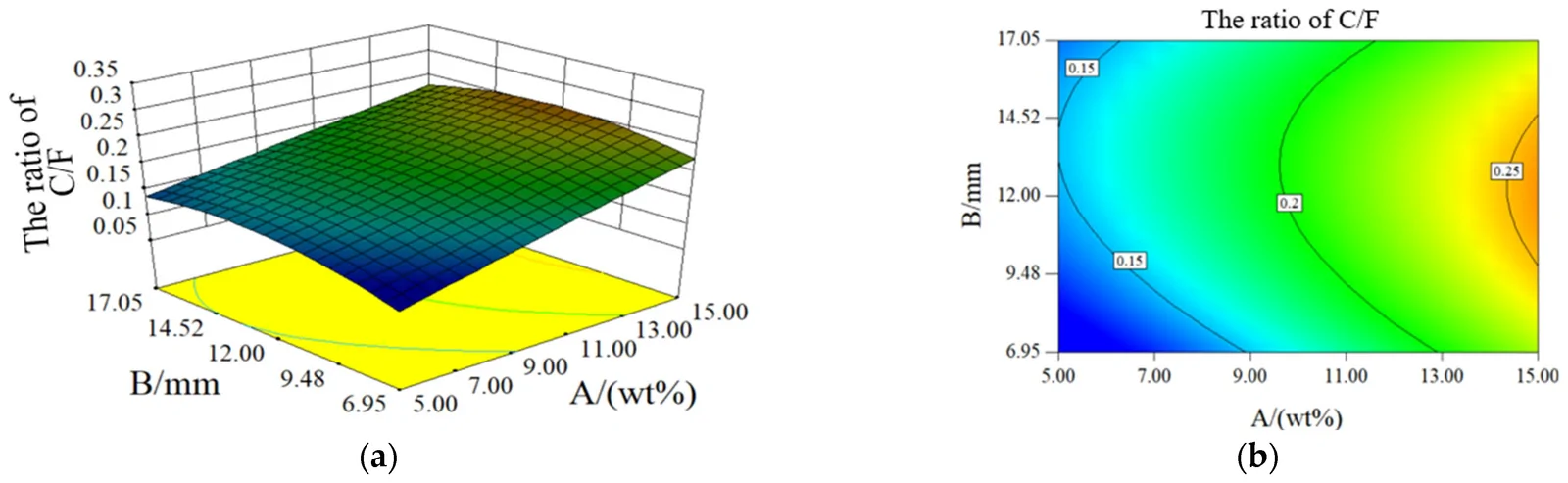

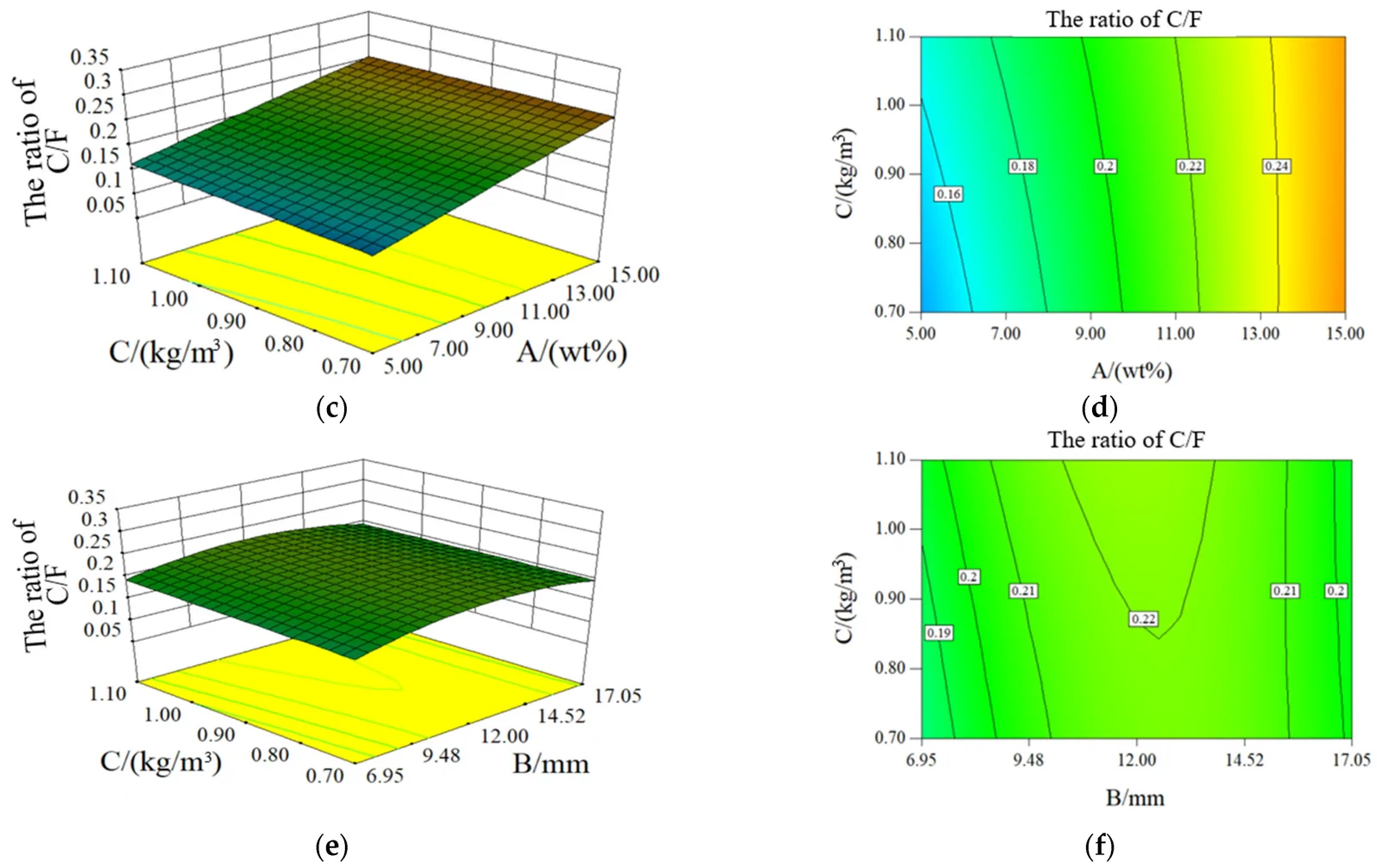
3D response surface (**a**) and contour plots (**b**) of flexural-to-compressive strength ratio: effects of emulsion content A and fiber length B at fixed fiber content C = 0.9 kg/m^3^; 3D response surface (**c**) and contour plots (**d**) of flexural-to-compressive strength ratio: effects of A and C at fixed B = 12 mm; 3D response surface (**e**) and contour plots (**f**) of flexural-to-compressive strength ratio: effects of B and C at fixed A = 10 wt.%.

The response surface curves for BC and AB in Figure 3 reveal that the flexural-to-compressive strength ratio reaches a peak value within the experimental factor range. When fiber length remains constant, increasing emulsion content initially correlates with an increased flexural-to-compressive strength ratio, which subsequently decreases after reaching a maximum at the optimal emulsion dosage. Consequently, the flexural-to-compressive strength ratio isoline appears elliptical, indicating that different fiber lengths correspond to different optimal emulsion dosages. Furthermore, the appropriate emulsion dosage for maximizing the flexural-to-compressive strength ratio decreases as fiber length decreases. The contour lines’ distribution along the abscissa is more pronounced than along the ordinate, suggesting that emulsion A exerts a greater influence than fiber length C and fiber content B. Based on formula 2 and Figure 3, the significant order of influence in the flexural-to-compressive strength ratio model is emulsion A > fiber content C > fiber length B, while the significant order of interaction among factors is AB > BC > AC. As shown in Figure 2c,e, flexural strength shows an approximately increasing trend with fiber content within the investigated range, which can be attributed to the enhanced crack-bridging effect of fibers [25,26]. Similar fiber-content-dependent improvements in flexural and post-cracking behavior have also been reported for steel fiber-reinforced and hybrid fiber-reinforced concrete.

The flexural-to-compressive strength ratio of FRPC reflects the material’s flexibility. A smaller ratio indicates greater brittleness, while a larger ratio signifies enhanced crack resistance and flexibility [26,27]. FRPC with superior flexibility is particularly valuable for reinforcing mine tunnels as it can effectively seal surrounding rock to prevent weathering, provide support, fill rock voids, and distribute external forces. The flexural-to-compressive strength ratio serves as a more reliable indicator of mining concrete flexibility than individual compressive and flexural strength measurements. Figure 3 shows that the flexural-to-compressive strength ratio is affected by the coupled effects of the three variables, and an optimal fiber length exists within the investigated range. Based on the desirability function, the optimal formulation was identified as 10% styrene–acrylic emulsion, 12 mm fiber length, and 1.1 kg/m^3^ fiber content. Verification tests were then conducted using this formulation, and the measured flexural-to-compressive strength ratio was close to the predicted value, confirming the reliability of the optimization result within the selected experimental domain. Future work should include more performance indicators, such as durability, permeability, workability, and fracture energy, to develop a more comprehensive optimization framework for FRPC.

### 3.4. Mechanical Characteristics of FRPC

**Figure 4:**
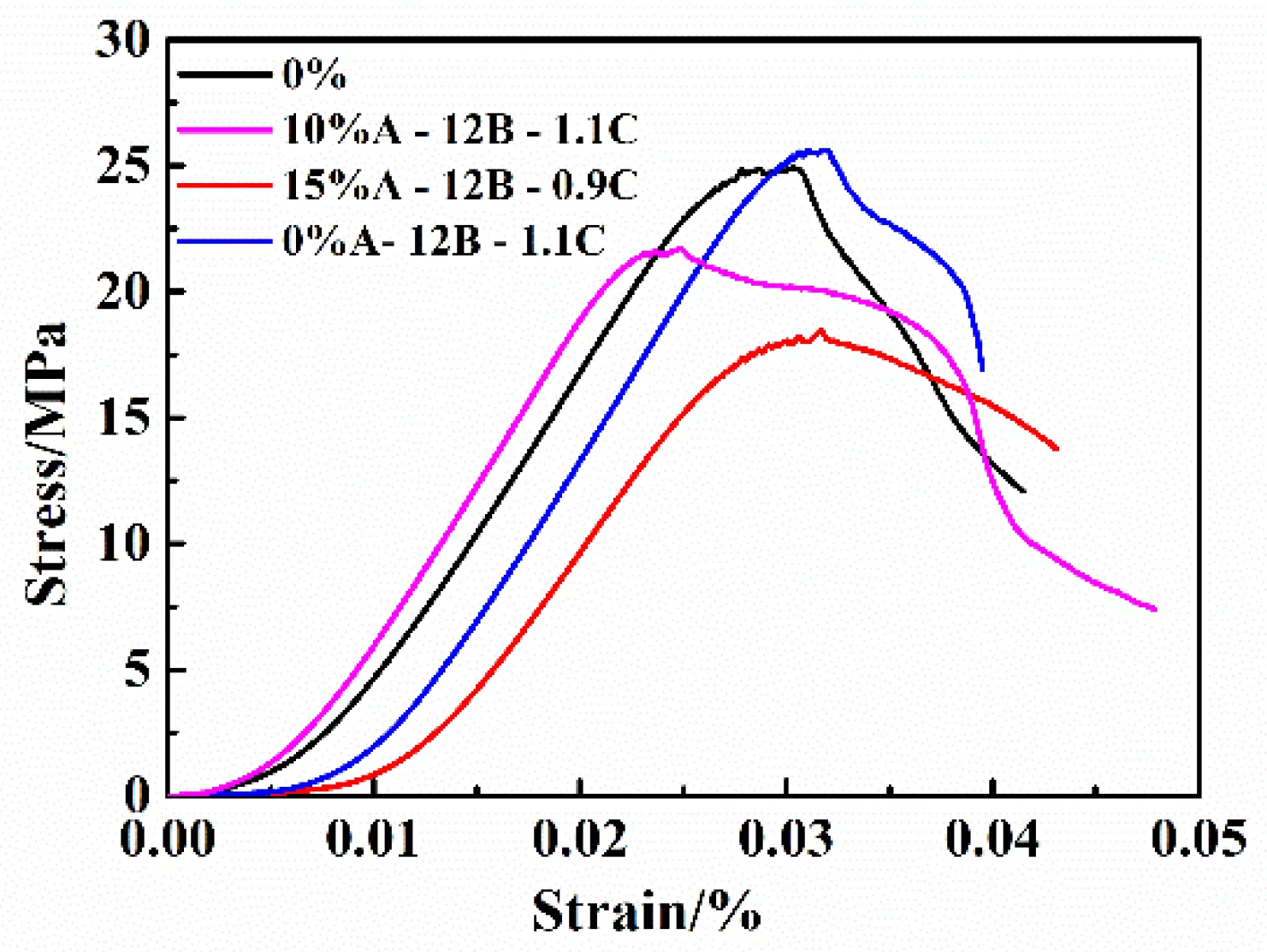
Stress–strain curves of FRPC.

This study evaluates the flexibility of FRPC through analysis of stress–strain curves under various emulsion and fiber content conditions. Fiber length was found to have a limited influence on the mechanical properties of FRPC. The test block’s flexibility was assessed by examining its post-compression failure state. Figure 4 illustrates the effects of varying emulsion and fiber content, where the control group (0%) contains neither emulsion nor fiber, while groups 0% A, 10% A, and 15% A correspond to increasing emulsion content. Group 12B contains fibers of specific length, and groups 0.9C and 1.1C represent different fiber contents. As demonstrated in Figure 4, the incorporation of emulsion and fiber reduces the concrete’s stress under maximum pressure, resulting in cracking without complete fracture or disintegration. This behavior can be attributed to the combined effect of emulsion and fibers with low elastic modulus, which facilitates energy accumulation during compression. The observed stress response and failure characteristics satisfy the flexibility requirements for mine concrete applications, effectively reducing concrete cracking and detachment in anchor spray protective layers, thereby preventing coal spontaneous combustion caused by air leakage channels.

**Figure 5:**
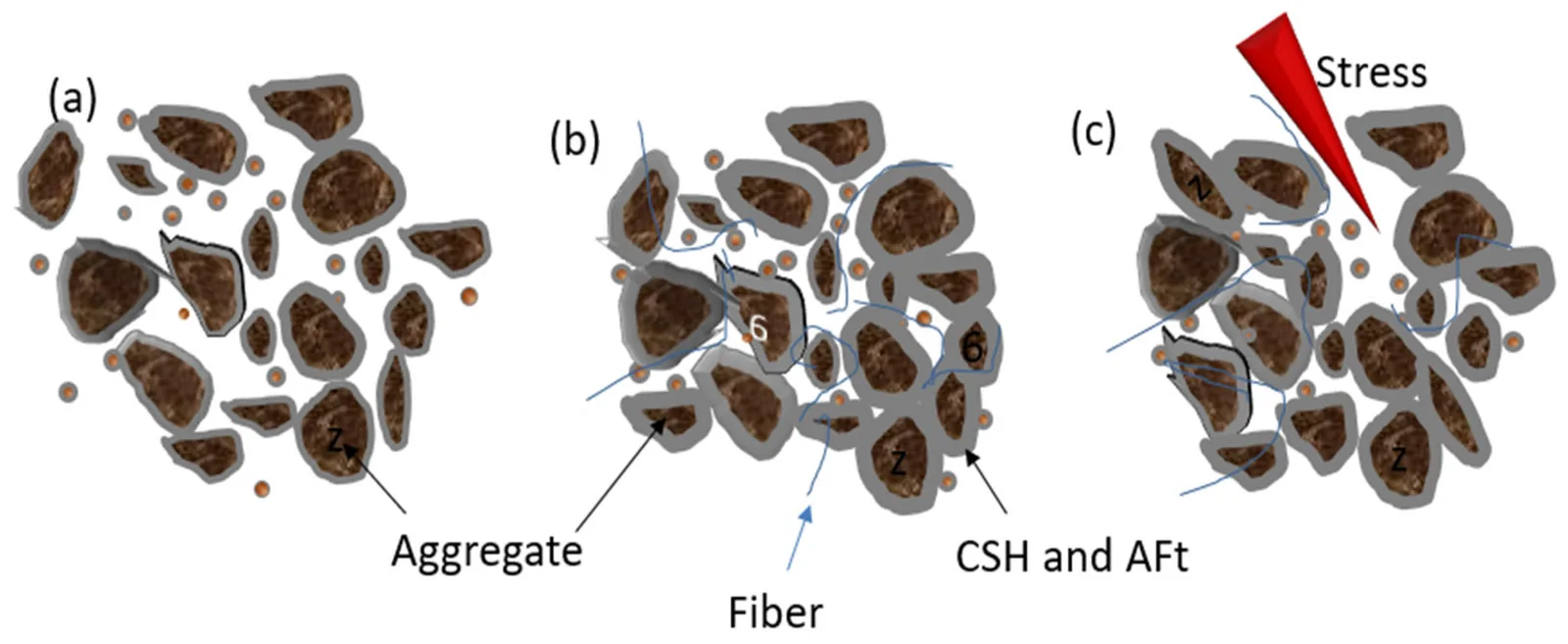
Schematic diagram of FRPC: (**a**) cement concrete, (**b**) cement concrete added emulsion and fiber (FRPC), (**c**) Stress failure diagram of FRPC.

Based on the stress failure state analysis, a schematic representation of a concrete structure with fiber and emulsion additions was developed (Figure 5). In Figure 5a, the cement concrete appears dispersed, whereas in Figure 5b, the addition of emulsion and fiber results in a more compact test block. The incorporation of fiber inhibits the formation and propagation of microcracks during loading due to its crack-bridging effect. Additionally, the bonding properties of the emulsion enhance concrete toughness, as illustrated in Figure 5c. However, excessive fiber content leads to intertwining and consolidation of polypropylene fibers, creating numerous voids and large cracks within the concrete, thereby reducing compactness and increasing internal weak interfaces. This phenomenon explains the decrease in mechanical properties observed in Section 2.2 and Section 2.3 when fiber content exceeds optimal levels.

### 3.5. Microstructure of FRPC

**Figure 6:**
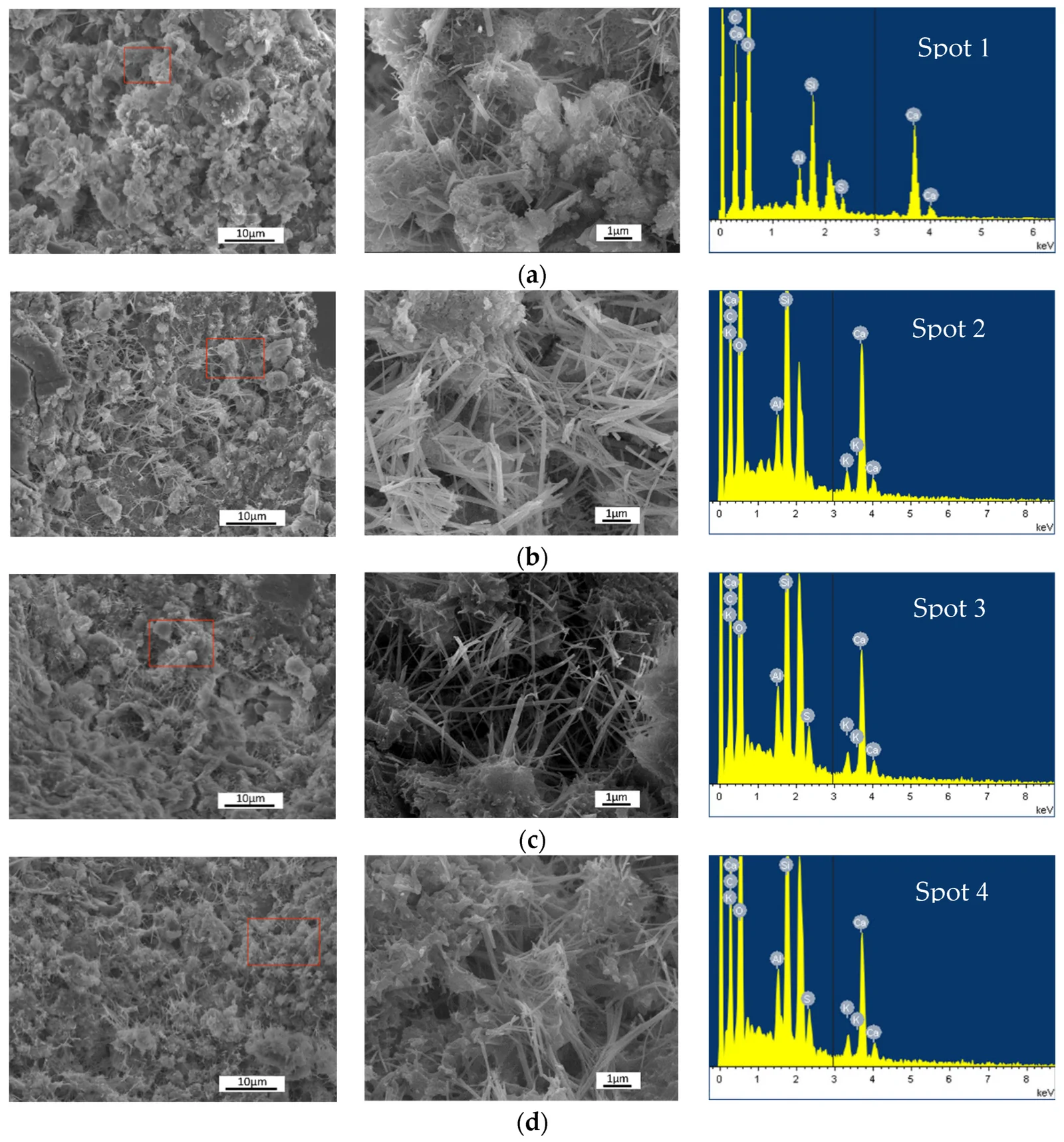

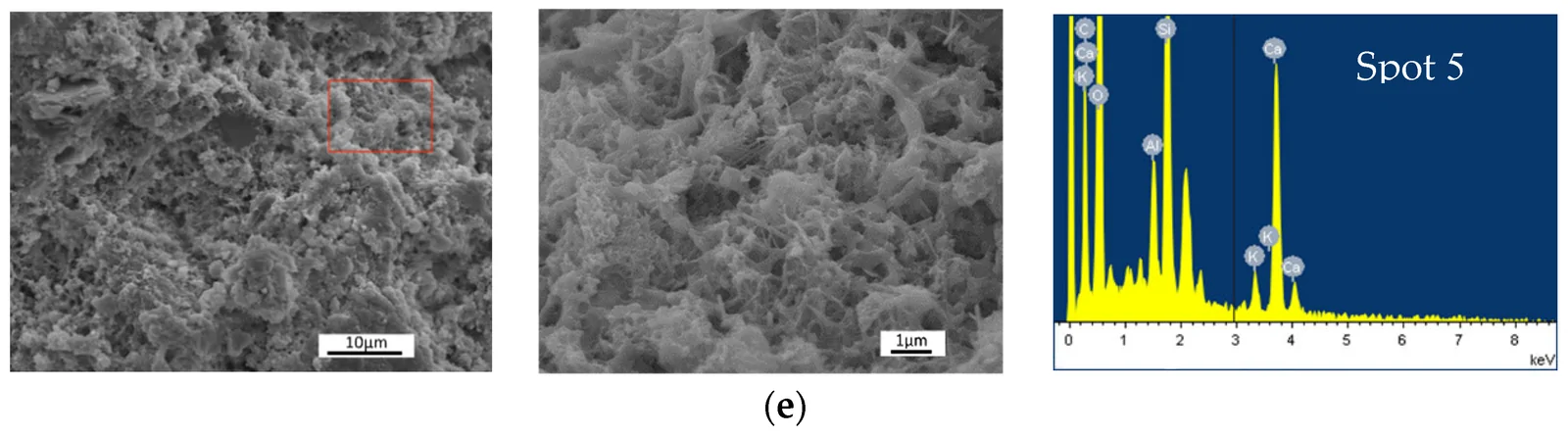
SEM-EDS analyses on FRPC with different emulsion additions: (**a**) 0%, (**b**) 5%, (**c**) 10%, (**d**) 15%, (**e**) 18.4%. The red box represents the spectral spot.

The SEM research institute conducted a 7-day cure of an experimental sample under standard conditions. The sample had a fiber length of 12 mm and an added amount of 1.1 kg/m^3^, with an EDS of ×10,000 surface scans (with a scale of 1 μm). The experimental results, presented in Figure 6, reveal that FRPC hydration products include calcium–silicate–hydrate (C-S-H), ettringite, dispersed flake-shaped Ca(OH)_2_, and calcium carbonate, which exhibit needle, long rod, flower cluster, and flake morphologies, respectively [28,29]. In Figure 6a, cement particles and hydration products are clearly distinguishable, while Figure 6b,c displays hydration products with distinctive morphological features. With the addition of emulsion, Figure 6d,e shows hydration products, self-aggregates of emulsion particles, and adsorption clusters formed between emulsion and cement. The electron microscope images demonstrate that with increasing emulsion content, ettringite, Ca(OH)_2_, and C-S-H aggregate more densely, enhancing the material’s impermeability. The EDS analysis results in Table 7 indicate that the addition of emulsion progressively reduces the C element content, thereby decreasing the amount of calcium carbonate in the product. The atomic ratio of Ca/Si and the proportion of O element increase, suggesting that emulsion addition inhibits the hydration and carbonization processes of C_3_S β-C_2_S, and C_3_A to varying degrees [30].

**Figure 7:**
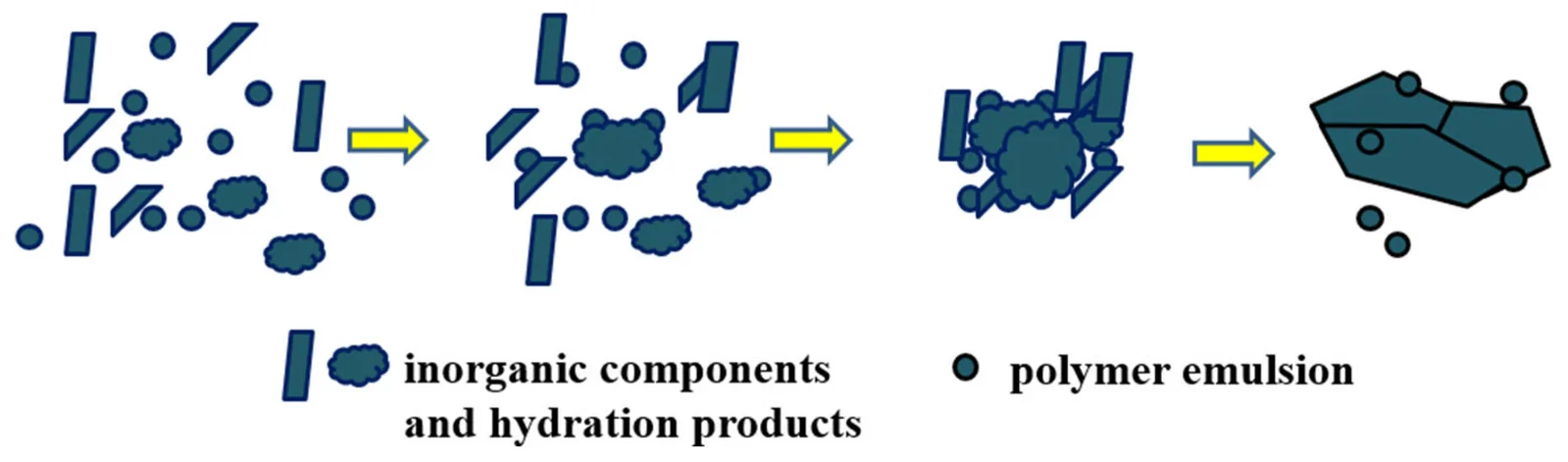
Schematic representation of polymer emulsion film formation.

According to the models proposed by Ohama and Beeldens [31,32], emulsion particles initially settle on cement and aggregate surfaces after mixing with concrete. As hydration progresses, these particles migrate into the pores. With decreasing free water, the particles form clusters and create a continuous membrane network structure. In this study, anionic styrene–acrylic emulsion adsorbs onto cement and aggregate surfaces through electrostatic attraction. Additionally, some polymers adsorb onto cement hydration product surfaces, neutralizing the local negative charge of cementitious materials. The flocculation effect of emulsion particles correlates with the amount added, with optimal flocculation occurring when positive and negative charges are balanced. This phenomenon explains the extreme values observed in the flexibility response surface diagram of FRPC in Section 2.3 and the decrease in compressive strength with emulsion addition described in Section 2.1. With appropriate emulsion content, a continuous film forms between emulsion particles and cement products, while self-aggregation and complexation of emulsion particles enhance structural compactness. The polymer film adheres to aggregate surfaces, improving bonding capability, interfacial performance, and fracture resistance of cement-based materials. This creates a more cohesive system with enhanced flexibility and impermeability, effectively preventing cracking and detachment of underground concrete and the formation of air leakage channels, ultimately reducing coal spontaneous combustion risk [17,18]. Figure 7 illustrates this mechanism.

### 3.6. Engineering Applications

The field application was conducted to demonstrate the practical feasibility of the optimized FRPC mixture under actual mining roadway conditions. The systematic effects of styrene–acrylic emulsion content, fiber length, and fiber content on FRPC performance were evaluated in Section 3.1, Section 3.2 and Section 3.3 based on the experimental and RSM results. Therefore, this section focuses on the field performance of the optimized mixture, including spraying thickness, surface cracking, detachment, and sealing behavior.

**Figure 8:**
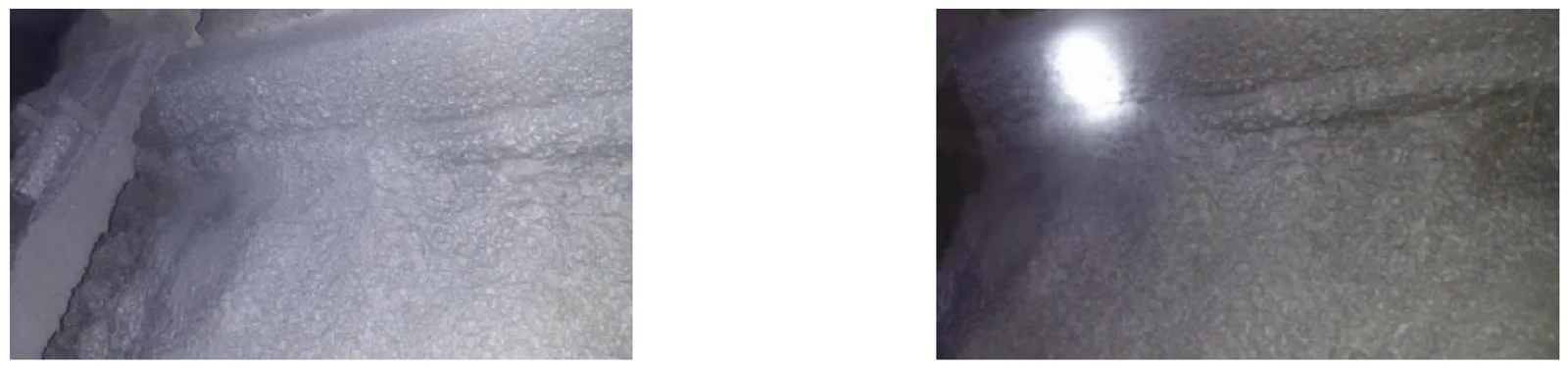
Schematic representation of polymer emulsion film formation.

The complex geological structure of the Dabaoding Coal Mine, resulting from tectonic movements and magmatic activity, has created safety concerns related to coal spontaneous combustion. Traditional support methods such as concrete spraying and anchoring nets have proven problematic due to mine pressure, deformation, cracking, and material detachment. To address these issues, FRPC was applied to the floor of coal seam 32 in the east wing of +1220 m horizontal secondary mining. The tunnel extends 469.7 m with a 10-year design service life. The roadway support system incorporates bolting and shotcreting, utilizing Fried Dough Twists resin anchor bolts at 1.0 m intervals with a concrete strength grade of C20. FRPC shotcrete containing 10% emulsion, 12 mm fiber length, and 1.1 kg/m^3^ fiber content was spray-applied over the anchor mesh. The spraying effect diagrams are shown in Figure 8. The evaluation of this method’s effectiveness focused on spraying thickness, appearance, cracking degree, and flexibility.

The FRPC spraying layer requires only 15 mm thickness, compared to the 20 mm needed for traditional concrete. With FRPC, both cracking risk and affected area have been significantly reduced, with minimal surface detachment observed. The flexural-to-compressive strength ratio of FRPC is 0.20, demonstrating superior material flexibility compared to ordinary concrete’s flexural-to-compressive strength ratio of 0.11 (as shown in Table 8). As shown in Table 8, compared with ordinary concrete, the optimized FRPC reduced the spraying thickness from 20 mm to 15 mm, decreased the number of cracks per unit area from 20 to 8 cracks/m^2^, and reduced the crack area per unit area from 146 to 50 mm^2^/m^2^. These correspond to reductions of 25%, 60%, and approximately 65.8%, respectively. Meanwhile, the flexural-to-compressive strength ratio increased from 0.11 to 0.20, indicating improved relative flexibility. These quantitative results demonstrate the potential of the optimized FRPC mixture for reducing cracking, detachment, and air leakage pathways in mining roadway support.

However, this field comparison should be regarded as a practical demonstration of the optimized mixture rather than a complete validation of the optimization methodology. Further field-scale tests involving different emulsion contents, fiber lengths, and fiber contents are still required to systematically evaluate the engineering performance of FRPC under different mining roadway conditions.

## 4. Conclusions

In response to improve the performance of cement-based materials, RSM analysis is used to establish a well-fitted model for 28-day compressive strength, flexural strength, and flexural-to-compression strength ratio of FRPC using styrene–acrylic emulsion, fiber length, and fiber content as variables. Results demonstrated that styrene–acrylic emulsion addition was the primary factor influencing FRPC’s performance. when using the flexural-to-compression strength ratio of FRPC as the flexibility index while maintaining the strength requirement of 20 MPa, the significance order of factors is: styrene–acrylic emulsion (A) > fiber content (C) > fiber length (B). The optimal formulation includes 10% styrene–acrylic emulsion, 12 mm fiber length, and 1.1 kg/m^3^ fiber content. Fiber and emulsion incorporation enhances FRPC’s flexibility, inhibits crack formation, and improves ductility under crushing loads. Appropriate emulsion addition improves FRPC’s flexibility and impermeability by forming a more cohesive system between cement and aggregate through adsorption and complexation mechanisms. The field demonstration in Dabaoding Coal Mine showed that the optimized FRPC mixture reduced surface cracking and detachment compared with ordinary concrete, indicating its potential for roadway support and sealing.

## Figures and Tables

**Table 1:** Experimental result of sand gradation.

Diameter/mm	4.75	2.36	1.18	0.6	0.3	0.15	<0.15
Sieve residue/%	0	0	12.5	38.8	31.3	13.2	4.2
Cumulative sieve residue/%	0	0	12.5	51.3	82.6	95.9	100

**Table 2:** Factors and levels in RSM.

Level	Factors		
	A/(wt.%)	B/mm	C/(kg/m^3^)
High	15	15	1.1
0	10	12	0.9
Low	5	9	0.7
r	18.40896	17	1.23636
−r	1.59103	7	0.56364

Note: The length of fiber in this experiment is an integer due to production difficulties in manufacturing 17.04538 mm and 6.95462 mm fibers.

**Table 3:** RSM design points and corresponding responses.

No.	A/wt.%	B/mm	C/(kg/m^3^)	Compressive Strength/MPa	COV/%	Flexural Strength/MPa	COV/%	Flexural-to-Compressive Strength Ratio
1	5	9	0.7	22.0	2.57	2.7	15.71	0.12
2	18.408 96	12	0.9	16.5	5.14	4.7	6.02	0.28
3	15	15	0.7	17.8	5.56	4.4	3.21	0.25
4	5	15	1.1	23.0	6.76	3.6	11.79	0.16
5	10	12	0.9	20.9	10.15	4.3	9.87	0.21
6	15	9	0.7	17.4	4.88	4.4	6.42	0.25
7	15	9	1.1	17.6	8.04	4.4	9.64	0.25
8	10	17	0.9	21.0	6.73	3.8	11.17	0.18
9	15	15	1.1	18.0	2.36	4.4	6.43	0.25

*Note: The compressive strength and flexural strength values listed in Table 3 are average values calculated from two repeated experimental tests for each mixture and were used for response surface modeling.” COV/% was calculated from the two replicate test results and is provided as a preliminary indicator of experimental variability.*

**Table 4:** The variance analysis of compressive strength.

Source	*f*-Value	*p*-Value	Statistical Significance
Model	68.77	<0.0001	Significant
A	593.99	<0.0001	
B	15.28	0.0016	
C	3.65	0.1340	
AB	0.22	0.6503	
AC	0.079	0.7849	
BC	0.0087	0.9274	
A^2^	2.25	0.1645	
B^2^	3.50	0.0909	
C^2^	0.40	0.5434	
Lack of fit	2.34	0.1862	Not significant

**Table 5:** The variance analysis of flexural strength.

Source	*f*-Value	*p*-Value	Statistical Significance
Model	69.45	<0.0001	Significant
A	447.44	<0.0001	
B	30.11	0.0003	
C	16.30	0.0024	
AB	11.42	0.0070	
AC	11.42	0.0070	
BC	3.53	0.0899	
A^2^	20.58	0.0011	
B^2^	90.75	<0.0001	
C^2^	0.067	0.8008	
Lack of fit	2.13	0.2134	Not significant

**Table 6:** The variance analysis of the flexural-to-compressive strength ratio.

Source	*f*-Value	*p*-Value	Statistical Significance
Model	111.68	<0.0001	Significant
A	939.16	<0.0001	
B	14.21	0.0030	
C	9.26	0.0030	
AB	20.38	0.0425	
AC	1.08	0.0425	
BC	2.91	0.2722	
A^2^	36.75	0.4338	
B^2^	151.43	<0.0001	
C^2^	10.82	0.7797	
Lack of fit	1.78	0.2718	Not significant

**Table 7:** The elemental atomic ratios of spots 1–5 in Figure 6.

Element	Spot 1/(at%)	Spot 2/(at%)	Spot 3/(at%)	Spot 4/(at%)	Spot 5/(at%)
O	50.83	61.45	61.87	59.68	63.60
C	32.24	21.44	21.90	16.51	16.74
Si	4.95	8.86	7.60	9.6	8.43
Ca	9.36	6.61	5.89	10.34	8.59
Al	1.52	0.98	1.19	1.91	1.61
S	–		0.81	0.9	
K		0.77	0.74	1.16	1.03
Total	100	100	100	100	100

**Table 8:** Comparison of material application effects in engineering.

Parameters	Traditional Concrete	FRPC
Spraying thickness/mm	20	15
Appearance	Shedding, cracking	Fewer shedding
Number of cracks per unit area (cracks/m^2^)	20	8
Crack area per unit area (mm^2^/m^2^)	146	50
Flexibility (flexural-to-compressive strength ratio)	0.11	0.20

## Data Availability

The original contributions presented in this study are included in the article. Further inquiries can be directed to the corresponding authors.
